# Solitary Lumbar Extradural Plasmacytoma With Minimal Vertebral Involvement: A Rare Presentation

**DOI:** 10.7759/cureus.101661

**Published:** 2026-01-16

**Authors:** Niraj K Choudhary, Dattatraya Mallik, Jeevesh Mallik, Manoj Kumar

**Affiliations:** 1 Neurosurgery, Tata Main Hospital, Jamshedpur, IND

**Keywords:** extradural spinal tumor, lumbar spine, plasma cell neoplasm, solitary plasmacytoma, spinal plasmacytoma

## Abstract

Solitary plasmacytoma of the spine is an uncommon plasma cell neoplasm that typically arises from the vertebral body and is associated with osteolytic changes. Presentation as a predominantly extradural mass with minimal osseous involvement is rare and poses a diagnostic challenge due to nonspecific imaging features. We present the case of a 52-year-old female who presented with severe low back pain and bilateral radiculopathy without motor deficit or sphincter dysfunction. MRI revealed a well-defined, homogeneously enhancing anterior extradural mass at the L5 level, causing significant thecal sac compression. Whole-body ¹⁸F-fluorodeoxyglucose PET-CT demonstrated a small focal lytic lesion involving the superior endplate of L5, without evidence of additional skeletal or extramedullary disease. The patient underwent surgical decompression with gross total excision. Histopathology and immunohistochemistry confirmed plasmacytoma. Systemic evaluation revealed monoclonal gammopathy with minimal marrow plasma cell infiltration and absence of CRAB features, consistent with a solitary spinal plasmacytoma with minimal vertebral involvement. This case highlights the importance of considering plasmacytoma in the differential diagnosis of enhancing epidural spinal lesions and emphasizes the role of surgery for diagnosis and decompression, followed by long-term surveillance due to the risk of progression to multiple myeloma.

## Introduction

Plasma cell neoplasms represent a spectrum of disorders ranging from localized tumors to disseminated systemic disease, with multiple myeloma being the most common entity [1]. Solitary plasmacytoma is a localized clonal proliferation of plasma cells and is classified as either solitary plasmacytoma of bone or solitary extramedullary plasmacytoma [1,2]. Involvement of the spine is well recognized; however, most spinal plasmacytomas arise from the vertebral body and are associated with osteolytic destruction and epidural extension [2,3]. Presentation as a predominantly extradural lesion with minimal or absent osseous involvement is rare and may mimic other epidural pathologies, such as lymphoma or metastatic disease [4,5].

Definitive diagnosis requires histopathological confirmation with immunohistochemistry, along with comprehensive systemic evaluation to exclude multiple myeloma in accordance with International Myeloma Working Group criteria [1,6]. We present a rare case of a lumbar spinal plasmacytoma presenting primarily as an extradural mass with minimal vertebral endplate involvement.

## Case presentation

A 52-year-old female presented with a one-month history of severe low back pain radiating to both lower limbs, refractory to conservative management, and associated with paresthesia for one week. There was no history of trauma, fever, weight loss, motor weakness, or bowel or bladder dysfunction. Her medical history was significant for hypertension, diabetes mellitus, and hypothyroidism, all well controlled with regular treatment.

On examination, the patient was alert and oriented. Neurological evaluation revealed normal motor strength, intact sensation, preserved deep tendon reflexes, and no signs of myelopathy. The straight leg raise test was positive bilaterally, reproducing radicular pain.

**Figure 1:**
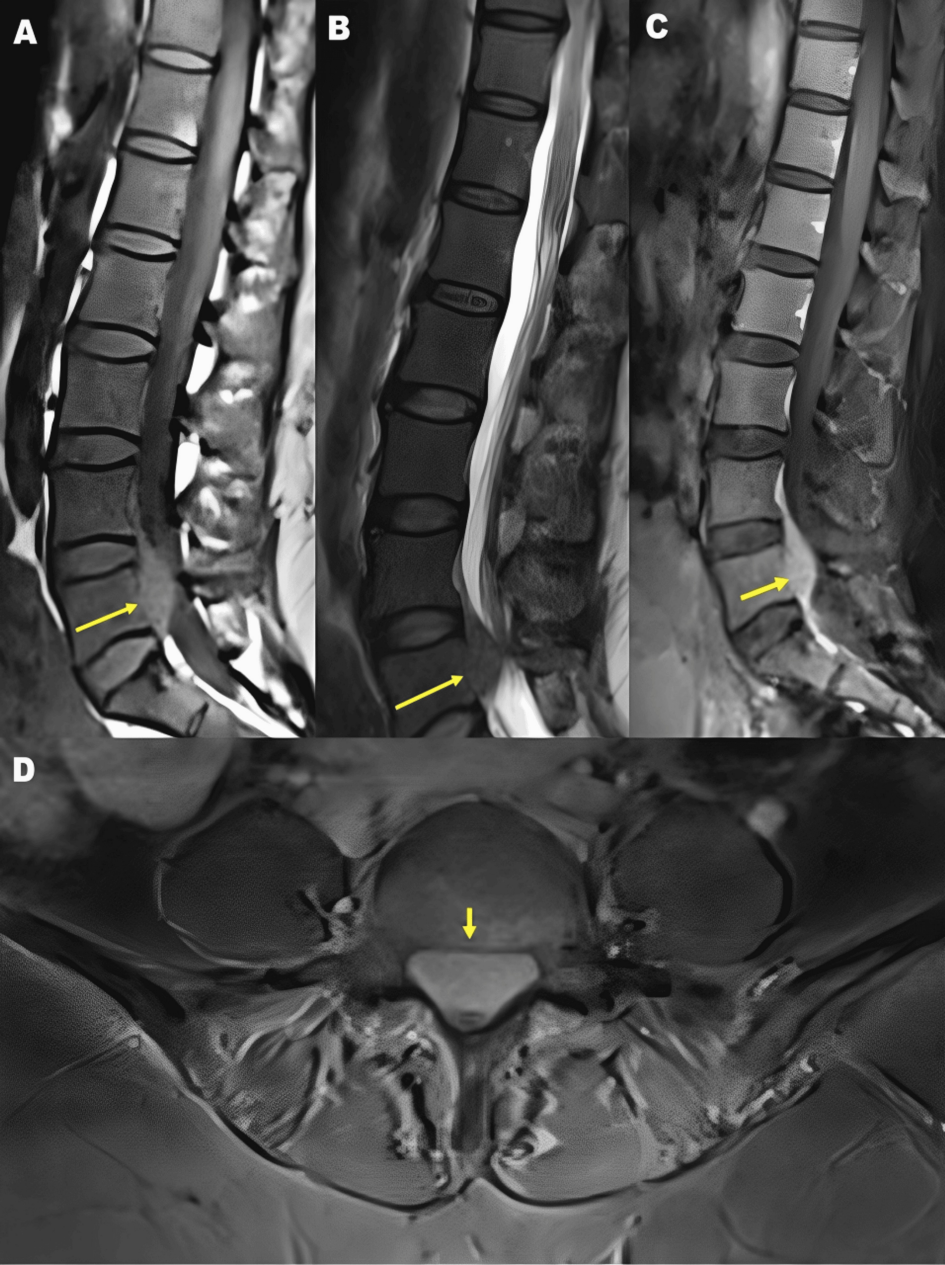
(A) Sagittal T1-weighted MRI of the lumbosacral spine at L5 showing an isointense anterior epidural lesion causing posterior displacement of the thecal sac. (B) Sagittal T2-weighted MRI at L5 demonstrating the lesion with mild hyperintensity and persistent thecal sac compression. (C) Sagittal post-contrast T1-weighted MRI showing avid enhancement of the anterior epidural lesion. (D) Axial post-contrast T1-weighted MRI at L5 demonstrating a homogeneously enhancing anterior extradural mass with thecal sac compression.

MRI of the lumbosacral spine demonstrated a well-defined anterior epidural soft-tissue lesion at the L5 vertebral level, causing posterior displacement and compression of the thecal sac. On sagittal T1-weighted images, the lesion appeared isointense (Figure 1A). Sagittal T2-weighted images showed the lesion to be mildly hyperintense, with persistent thecal sac compression (Figure 1B). Post-contrast T1-weighted sagittal images revealed homogeneous enhancement of the epidural lesion without obvious paraspinal soft-tissue extension (Figure 1C). An axial post-contrast T1-weighted image at the L5 level further demonstrated an anterior extradural enhancing lesion with thecal sac compression (Figure 1D).

No definitive vertebral body marrow signal abnormality was identified on MRI, a finding reported in some cases of spinal extradural plasmacytomas but not disease-specific [4,7].

The patient subsequently underwent an L5 laminectomy with gross total excision of the anterior epidural lesion. Intraoperatively, the mass was soft, friable, mildly vascular, non-encapsulated, and easily separable from the dura. Postoperatively, the patient experienced significant improvement in radicular pain.

**Figure 2:**
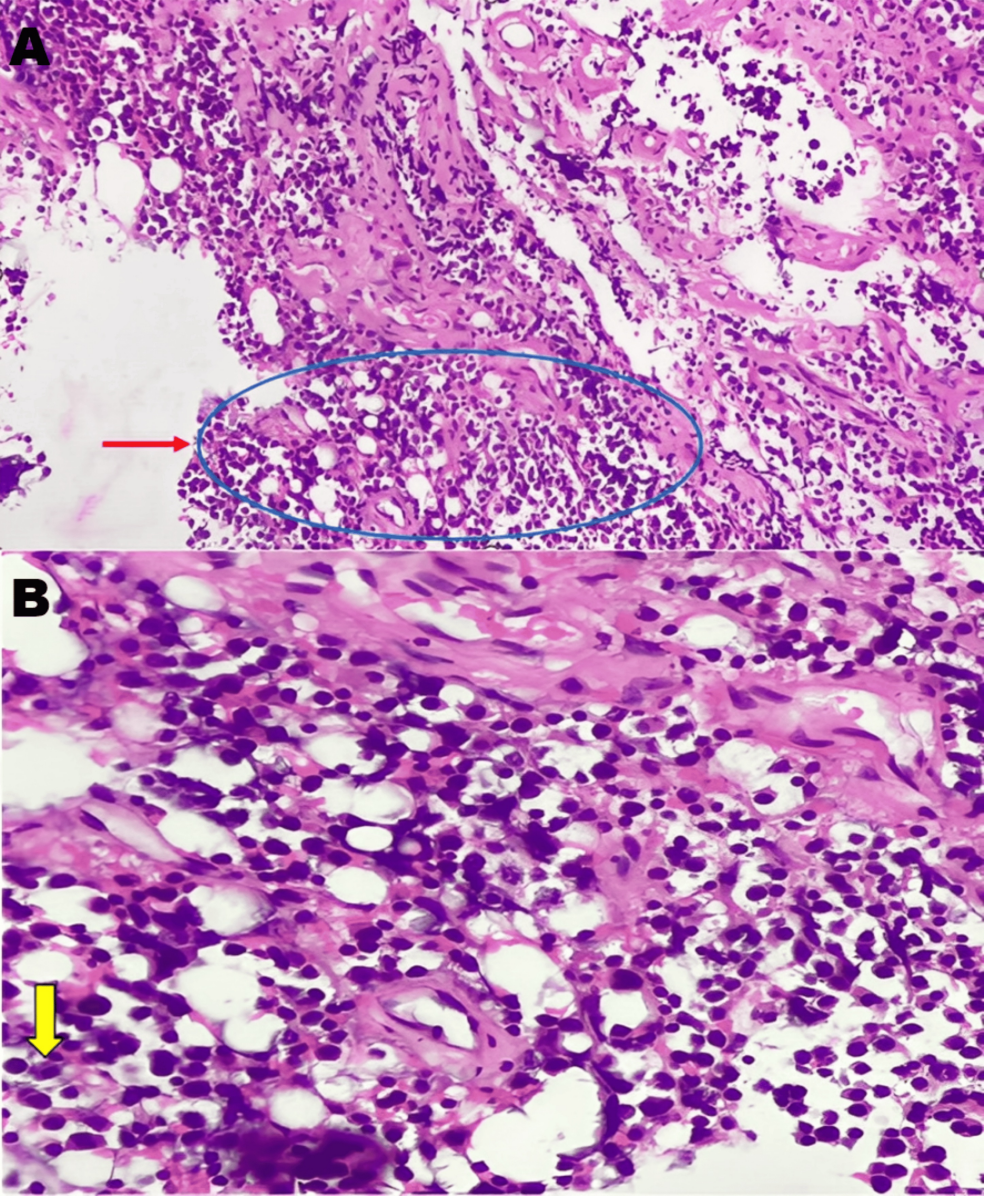
(A) Low-power photomicrograph (H&E stain, ×10) showing fibro-stromal tissue infiltrated by sheets and discohesive clusters of plasma cells admixed with mature lymphocytes. (B) High-power photomicrograph (H&E stain, ×400) demonstrating plasma cells with eccentrically placed nuclei, nuclear enlargement, coarse chromatin, basophilic cytoplasm, and occasional binucleate forms, consistent with plasma cell neoplasm.

Histopathological examination of the excised lesion revealed diffuse sheets of plasma cells infiltrating a fibro-stromal background (Figure 2A). At higher magnification, the tumor cells demonstrated eccentrically placed nuclei, coarse “clock-face” chromatin, abundant basophilic cytoplasm, and prominent perinuclear clearing, consistent with a plasma cell neoplasm (Figure 2B).

Immunohistochemistry showed diffuse positivity for CD138 and MUM-1 with kappa light chain restriction, confirming the diagnosis of a plasma cell neoplasm.

Further systemic evaluation revealed elevated serum free kappa light chains (75.25 mg/L) with lambda light chains of 10.85 mg/L, resulting in an abnormal kappa/lambda ratio of 6.935. Serum protein electrophoresis demonstrated a monoclonal spike in the gamma region. Immunoglobulin profiling showed elevated IgG with suppressed IgA and IgM levels. Urine examination for Bence Jones protein was negative.

**Figure 3:**
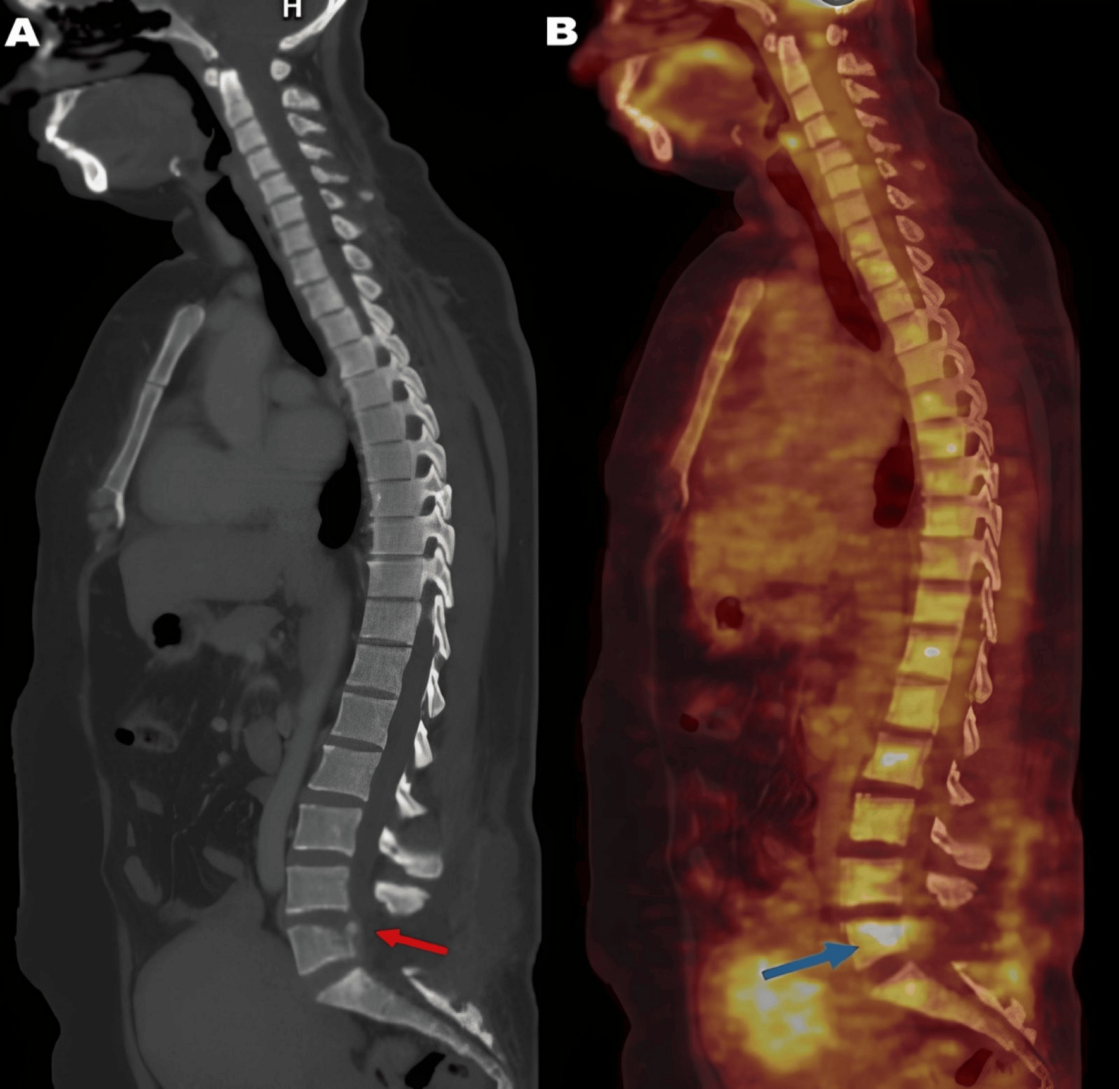
(A) Sagittal CT reconstruction of the lumbosacral spine demonstrating subtle cortical irregularity with minimal lytic erosion of the superior endplate of the L5 vertebral body. (B) Sagittal FDG PET-CT fusion image showing focal avid FDG uptake at the same site, with no additional hypermetabolic lesions.

Whole-body ¹⁸F-fluorodeoxyglucose PET-CT demonstrated postoperative changes at the L5 level. Sagittal CT reconstruction showed subtle cortical irregularity with minimal lytic erosion involving the posterior aspect of the superior endplate of the L5 vertebral body (Figure 3A). Corresponding sagittal FDG PET-CT fusion images demonstrated focal avid FDG uptake at the same site, with no evidence of additional hypermetabolic skeletal or soft-tissue lesions elsewhere, suggesting a localized disease process (Figure 3B).

Bone marrow examination revealed approximately 12% plasma cells without cytogenetic abnormalities, and fluorescence in situ hybridization was negative for high-risk clonal changes. In the absence of CRAB features, these findings favored a diagnosis of solitary spinal plasmacytoma with minimal vertebral involvement rather than multiple myeloma.

## Discussion

Solitary plasmacytoma is an uncommon plasma cell neoplasm, accounting for a small proportion of plasma cell dyscrasias, and frequently involves the axial skeleton, particularly the spine [1,2]. Most spinal plasmacytomas originate within the vertebral body and produce osteolytic destruction with secondary epidural extension; lesions presenting predominantly in the epidural space with minimal osseous involvement are distinctly uncommon [2,3].

Clinical manifestations are related to local mass effect and include axial pain, radiculopathy, and neurological deficits, depending on the level and degree of compression [5,8]. MRI is the modality of choice and typically demonstrates a well-defined, homogeneously enhancing epidural mass; however, these features overlap with those of lymphoma and metastatic disease, making preoperative diagnosis challenging [4,7]. PET-CT plays a crucial role in excluding additional skeletal lesions and identifying subtle vertebral involvement not always evident on MRI, as observed in the present case [1,6,9].

Definitive diagnosis requires histopathological confirmation with immunohistochemistry demonstrating monoclonal plasma cells, while systemic evaluation is essential to differentiate solitary plasmacytoma from multiple myeloma [1,6]. Radiotherapy is considered the standard treatment for solitary plasmacytoma due to excellent local control rates; however, surgical decompression is indicated in cases of significant neural compression or when tissue diagnosis is required [1,8]. Long-term follow-up is mandatory, as a significant proportion of patients eventually progress to multiple myeloma despite initially localized disease [1,9].

## Conclusions

Spinal plasmacytoma may rarely present as a predominantly extradural mass with minimal vertebral involvement, posing a diagnostic challenge. Recognition of this entity is important when evaluating homogeneously enhancing epidural spinal lesions. Surgical decompression provides neurological improvement and allows for definitive diagnosis. Comprehensive systemic evaluation and vigilant long-term surveillance are essential due to the risk of progression to multiple myeloma.
